# Window leakage in clinical EEG machine learning: matched subject-independent evaluation across ADHD and Parkinson cohorts

**DOI:** 10.3389/fninf.2026.1930795

**Published:** 2026-09-29

**Authors:** Burak Aggul

**Affiliations:** Department of Database, Network Design and Management, Plato Vocational School, Istanbul Topkapi University, Istanbul, Türkiye

**Keywords:** ADHD, clinical machine learning, cross-cohort replication, electroencephalography, Parkinson's disease, subject-independent validation, time-series evaluation, window leakage

## Abstract

Windowed electroencephalography (EEG) pipelines can produce misleadingly high performance when segments from the same participant occur in both training and testing. We define this contamination as *window leakage* and test it in two clinically and technically distinct public cohorts: 121 children (61 attention-deficit/hyperactivity disorder [ADHD], 60 controls) and 149 adults (100 Parkinson's disease, 49 controls). Signals were harmonized to 18 common channels and 128 Hz. All primary models used stratified 8-fold participant-disjoint evaluation, 50%-overlapping windows, participant-level probability aggregation, and validation participants drawn exclusively from each outer-training fold. A deliberately contaminated Random Forest audit contrasted random-window and participant-disjoint splitting. Random-window balanced accuracy reached 95.59% in ADHD and 92.02% in Parkinson's, but fell to 60.98% and 56.17%, respectively, when participants were held out. Thus, the inflation replicated across disorders, ages, recording tasks, and acquisition systems. Under correct evaluation, CNN-LSTM achieved the highest ADHD subject-level balanced accuracy (73.21%), whereas EEGNet achieved the highest Parkinson's balanced accuracy (63.75%). No architecture comparison remained significant after Bonferroni correction. One-, 2-, 4-, and 8-s windows produced no consistent cross-cohort ranking. Removing ATCNN-SA components also yielded dataset-dependent changes, and no ablation difference was significant after correction. Participant-bootstrap intervals remained wide despite tens of thousands of windows, indicating that inferential precision was governed by participant count rather than segment count. Window-level random splits contaminate clinical EEG performance estimates and must not be used to claim generalization to unseen individuals. Participants should be separated before model fitting, results should be reported at subject level, and split, windowing, aggregation, and uncertainty procedures should be disclosed explicitly.

## 1 Introduction

Clinical machine-learning studies often divide one physiological recording into many short windows. This increases the number of model inputs but does not increase the number of independent participants. If windows from one participant appear in both training and testing, a classifier may exploit stable person-, session-, or device-specific structure rather than disease-related information. We use *window leakage* for this contamination, including the especially severe case in which temporally overlapping windows from one recording cross the split boundary. Leakage is a recognized cause of irreproducible machine-learning claims (Kapoor and Narayanan, 2023). In translational EEG, failure to hold out participants can strongly inflate apparent generalization (Brookshire et al., 2024).

The statistical problem is equally important. Adjacent windows are correlated, repeated windows inherit a common participant label, and cross-validation training sets overlap. Window count is therefore not an independent sample size for confidence intervals or significance testing. Model selection must be separated from performance estimation (Varma and Simon, 2006). Small neuroimaging cohorts can retain large uncertainty even under correct participant-level cross-validation (Varoquaux, 2018). A defensible clinical EEG study consequently requires participant-disjoint splitting, subject-level outcomes, uncertainty procedures that preserve clustering, and complete disclosure of window generation and aggregation.

We investigate these requirements in deliberately different case studies. The first is pediatric EEG recorded during a visual counting task for ADHD, a disorder whose clinical assessment remains multimodal rather than EEG-defined (Wolraich et al., 2019). The second is adult resting-state EEG for Parkinson's disease. The cohorts differ in diagnosis, age, behavioral state, channel density, sampling rate, and class balance. Parkinson's data are not an external test set for an ADHD classifier because the diagnostic targets differ. Instead, the second cohort tests whether the same evaluation failure and the sensitivity to architecture and window duration recur in another clinical EEG setting.

The study has three objectives. First, it quantifies random-window inflation and tests whether it replicates across both cohorts. Second, under participant separation, it compares four neural architectures and four window durations using a matched protocol. Third, it evaluates subject-level aggregation, uncertainty, computational cost, and four ATCNN-SA component removals. The primary contribution is methodological. ATCNN-SA is one evaluated architecture; universal or statistically significant model superiority is not claimed.

## 2 Related work

Data leakage occurs when information unavailable at deployment influences model development or evaluation. In biomedical prediction, repeated measurements and clustered observations make this risk particularly acute. Brookshire et al. found that participant-level holdout is frequently unclear in translational deep-learning EEG studies (Brookshire et al., 2024). Das et al. (2026)recently demonstrated pronounced performance reductions when models for the same pediatric ADHD cohort were moved from window-wise to subject-wise validation. These findings establish the split unit as a scientific design decision rather than an implementation detail.

EEG classifiers span hand-engineered spectral methods and end-to-end neural networks. EEGNet provides a compact convolutional architecture for multichannel EEG (Lawhern et al., 2018). Convolutional-recurrent networks combine local temporal filtering with sequence modeling (Zhang et al., 2018). Deep convolutional decoding introduced practical visualization approaches for learned EEG representations (Schirrmeister et al., 2017). EEG Conformer combines convolution with global attention (Song et al., 2023), whereas BENDR uses self-supervised representation learning across heterogeneous EEG datasets (Kostas et al., 2021). None of these architectural advances can correct a split that shares participants across development and evaluation.

ADHD EEG studies further illustrate sensitivity to representation and preprocessing. Quantitative EEG findings vary across age and clinical composition (Snyder and Hall, 2006), and EEG-defined subgroups are heterogeneous (Clarke et al., 2001). Earlier classification combined nonlinear features with neural networks (Mohammadi et al., 2016), whereas later work used subtype-specific convolutional models (Ahmadi et al., 2021). Garcia-Ponsoda et al. quantified the effect of preprocessing and temporal segmentation on pediatric EEG classification (García-Ponsoda et al., 2024). Recent studies have used topological search (Cai et al., 2025), graph convolution over multi-domain features (Li et al., 2025), convolutional frames (Hassan and Singhal, 2024), power-spectral-density maps (Latifi et al., 2024), and autoencoder-attention residual learning (Bansal et al., 2025). Their numerical results cannot be compared without an auditable account of participant separation.

Parkinson's EEG classification presents the same evaluation question under different signal conditions. Sugden and Diamandis emphasized generalization across Parkinson's cohorts (Sugden and Diamandis, 2023). A multi-center resting-state study likewise treated site variation as a central challenge (Kurbatskaya et al., 2023). The public cohort used here contains two minutes of eyes-open resting EEG for 100 individuals with Parkinson's disease and 49 controls (Singh et al., 2026); its associated study documents the clinical collection and cognitive context (Singh et al., 2023). The remaining gap is a matched demonstration of split, architecture, and window effects across distinct clinical cohorts while preserving the participant as the independent unit.

## 3 Methods

### 3.1 Cohorts, ethics, and shared preprocessing

The analysis used two public EEG cohorts chosen for complementary signal and clinical properties (Table 1). The *EEG Data for ADHD/Control Children* dataset was obtained from IEEE DataPort (DOI: 10.21227/rzfh-zn36) (Nasrabadi et al., 2020). The companion report describes 121 children aged 7–12 years, including 61 diagnosed with ADHD and 60 controls (Ekhlasi et al., 2021). EEG was acquired from 19 standard 10–20 positions at 128 Hz with A1/A2 references while children viewed cartoon images and counted characters. The source report states that the Institutional Review Board and Ethical Committee of Tehran University of Medical Sciences approved the procedures and that parents provided written informed consent; no approval identifier is reported.

**Table 1:** Cohort composition and matched analysis configuration.

Characteristic	ADHD cohort	Parkinson's cohort
Participants	121 children	149 adults
Cases / controls	61 / 60	100 / 49
Recording condition	Visual counting task	Resting state, eyes open
Source channels / sampling rate	19 / 128 Hz	63 / 500 Hz
Analyzed channels / sampling rate	18 / 128 Hz	18 / 128 Hz
Analyzed duration	Available task recording	120 s after a 5-s offset
Reference window / overlap	2 s / 50%	2 s / 50%
Retained 2-s windows	15,014	17,056

The Parkinson's cohort was the public NEMAR/OpenNeuro *Rest eyes open* dataset, OpenNeuro version 1.0.0 (DOI: 10.18112/openneuro.ds004584.v1.0.0) (Singh et al., 2026). It contains 149 adults, including 100 participants with Parkinson's disease and 49 controls, recorded for two minutes with eyes open. The released recordings contain 63 scalp channels sampled at 500 Hz. The associated study reports written informed consent and University of Iowa approval under IRB #201707828 (Singh et al., 2023). The present work was a secondary analysis of public, de-identified data and involved no recruitment, intervention, contact, or attempted re-identification.

The matched montage contained Fp1, Fp2, F7, F3, Fz, F4, F8, T7, C3, Cz, C4, T8, P7, P3, P4, P8, O1, and O2. Pz from the ADHD montage and channels without an exact cross-cohort counterpart were excluded. Each participant was processed independently. A fifth-order zero-phase Butterworth band-pass filter (0.5–40 Hz) was applied at the source sampling rate. Parkinson's signals were resampled to 128 Hz, and a 120-s segment beginning after a 5-s offset was retained. Each channel was standardized by its within-participant mean and standard deviation. No statistic was pooled across participants.

Signals were segmented into 1-, 2-, 4-, and 8-s windows with 50% overlap. A window was rejected when its maximum absolute standardized amplitude was 8 or greater. The 2-s reference retained 15,014 ADHD and 17,056 Parkinson's windows. The 1-, 4-, and 8-s settings retained 31,716, 6,754, and 2,768 ADHD windows and 34,860, 8,194, and 3,800 Parkinson's windows, respectively. Throughout the manuscript, *window* denotes a signal segment; *epoch* denotes one optimization pass.

Figure 1 summarizes the matched cross-cohort pipeline from signal harmonization and windowing to the two evaluation designs. Participant-disjoint splitting provided the leakage-free primary evaluation, whereas random-window splitting was retained solely as a deliberately contaminated audit.

**Figure 1:**
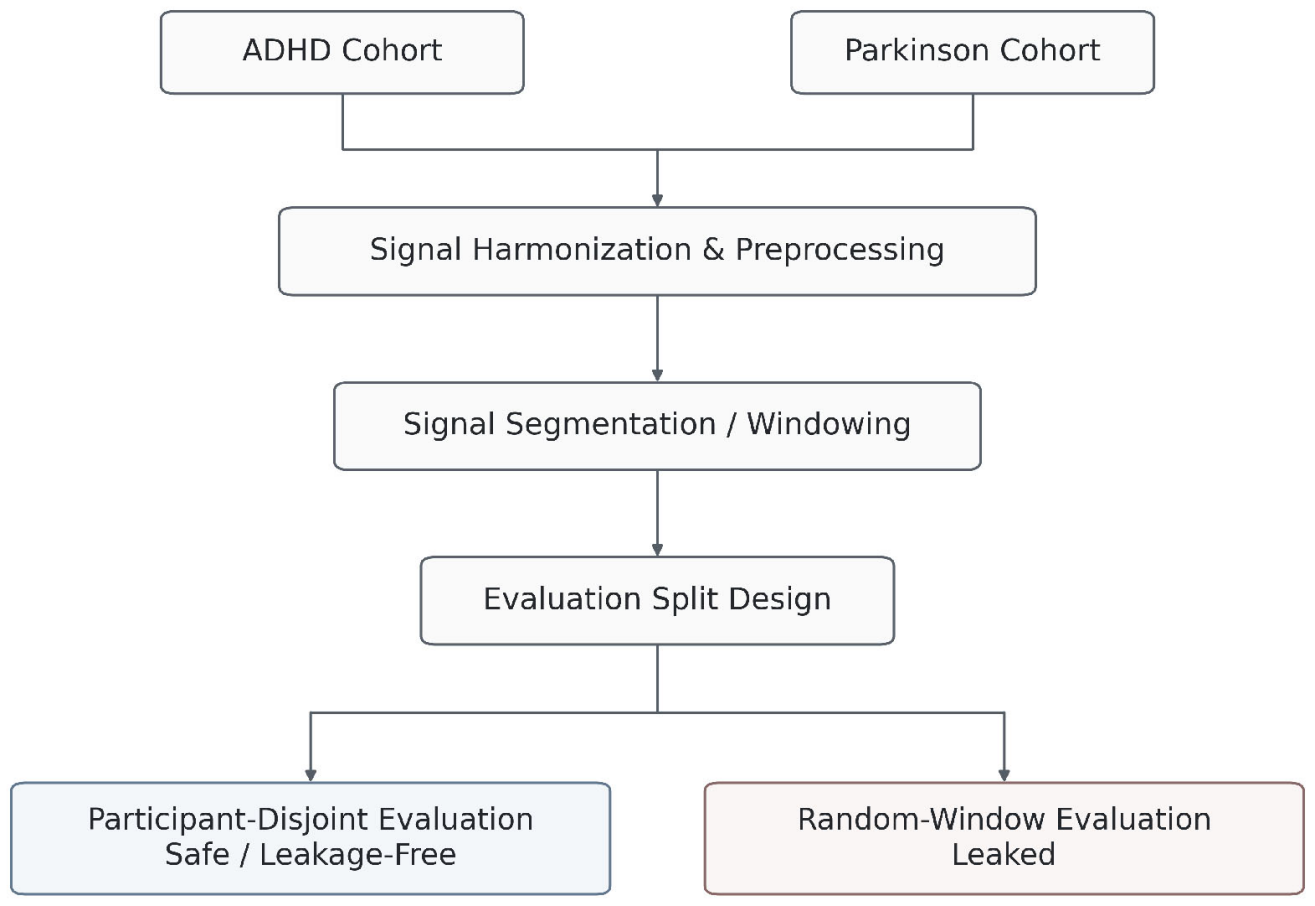
Cross-cohort methodology workflow. ADHD and Parkinson's EEG underwent matched signal harmonization and windowing before participant-disjoint (leakage-free) and random-window (leaked) evaluation.

### 3.2 Evaluation design and leakage audit

All inferential evaluation was participant-disjoint. Stratified 8-fold partitions were generated separately within each cohort using seed 20260903. Every participant appeared in exactly one outer test fold, and all windows from that participant remained in the same fold. Within each outer-training set, 15% of training participants were selected by a stratified split for validation, early stopping, learning-rate control, and training of the secondary subject aggregator. Outer-test participants were unavailable during every development decision. Eight folds were retained as a compromise between fold-level replication and clinically interpretable test-fold size. Five folds would provide only five paired observations, whereas ten folds would reduce each Parkinson's test fold to approximately 15 participants, including only four or five controls. The resulting eight paired observations still provide limited power, so all fold-wise tests were treated as descriptive. Robustness was assessed through independent-cohort replication and participant-level bootstrap intervals rather than by treating estimates from repeated, overlapping cross-validation training sets as independent evidence.

The primary audit compared this design with a deliberately contaminated stratified 80/20 random-window split. Random-window evaluation was repeated 10 times with seeds derived deterministically from the global seed. Because windows, rather than participants, were assigned, participant identities occurred on both sides of each random split. Preprocessing and feature extraction preceded both split strategies; the manipulated factor was the split unit.

Random Forest received 252 features per 2-s window. For each of 18 channels, Welch spectra provided absolute and relative delta (0.5–4 Hz), theta (4–8 Hz), alpha (8–13 Hz), beta (13–30 Hz), and gamma (30–40 Hz) power. Mean, standard deviation, mean absolute amplitude, and amplitude range supplied four time-domain features, giving 14 features per channel. The classifier used 300 trees, square-root feature sampling, balanced-subsample class weights, unlimited depth, and all available CPU threads, following the bootstrap-aggregation framework of Breiman (2001). The same feature extractor and classifier settings were used in both cohorts.

### 3.3 Model architectures

Four neural architectures received standardized *T*×18 windows. The 1D-CNN comprised three same-padded ReLU convolutions with 32, 64, and 128 filters and kernels 5, 5, and 3. The first two blocks included batch normalization, factor-2 max pooling, and dropout 0.3. Global average pooling, dropout 0.4, a 64-unit ReLU layer, dropout 0.3, and a two-unit softmax completed the model. CNN-LSTM used 64- and 128-filter convolutional blocks with kernels 5 and 3, followed by a 64-unit LSTM, a 64-unit ReLU layer, dropout 0.3, and softmax. Recurrent dropout was zero so the recurrent computation remained compatible with cuDNN. EEGNet used *F*_1_ = 8, a temporal kernel of 64, an 18 × 1 depthwise kernel with multiplier *D* = 2 and max-norm 1, 1 × 4 average pooling, a separable 1 × 16 convolution with *F*_2_ = 16, 1 × 8 average pooling, ELU activations, dropout 0.5, and a two-unit softmax head (Lawhern et al., 2018).

ATCNN-SA combined multi-scale convolution, feature-map recalibration, temporal weighting, and bidirectional recurrence. For a 2-s input **X** ∈ ℝ^256 × 18^, three parallel convolutional branches used kernels K={3,7,15} and 32 filters. The ATCNN-SA architecture used in this study is mathematically defined in Equations 1–6.


$$\begin{array}{l} {\text{H}}_{k}=\mathrm{ReLU}\left({\text{Conv1D}}_{k,32}\left(\text{X}\right)\right),\;k\in K. \end{array}$$ (1)

The branches were concatenated, batch-normalized, max-pooled, and regularized:


$$\begin{array}{l} {\text{H}}_{\text{ms}}={\text{Drop}}_{0.3}\left({\text{Pool}}_{2}\left[\text{BN}\left({\text{H}}_{3}∥{\text{H}}_{7}∥{\text{H}}_{15}\right)\right]\right). \end{array}$$ (2)

A 128-filter convolution with kernel 5 was followed by the same normalization, pooling, and dropout sequence:


$$\begin{array}{l} \text{H}={\text{Drop}}_{0.3}\left({\text{Pool}}_{2}\left[\text{BN}\left(\text{ReLU}\left({\text{Conv1D}}_{5,128}\left({\text{H}}_{\text{ms}}\right)\right)\right)\right]\right). \end{array}$$ (3)

Squeeze-and-excitation recalibrated the 128 learned feature maps (Hu et al., 2018). For *T*′ latent positions, $z_{c}={T^{\prime }}^{-1}\sum_{t=1}^{T^{\prime }}H_{t,\;c}$ and reduction ratio 8,


$$\begin{array}{l} \text{s}=\sigma \left({\text{W}}_{2}\text{ReLU}\left({\text{W}}_{1}\text{z}+{\text{b}}_{1}\right)+{\text{b}}_{2}\right),\;{\overset{\text{\textasciitilde{}}}{H}}_{t,c}=s_{c}H_{t,c}. \end{array}$$ (4)

These are feature-map weights rather than direct electrode weights. The temporal-attention layer assigned one additive score to each latent position (Bahdanau et al., 2015):


$$\begin{array}{l} e_{t}=\text{tanh}\left({\text{w}}_{a}^{⊤}{\tilde{\text{H}}}_{t}+b_{a}\right),\;\alpha_{t}=\frac{\exp\left(e_{t}\right)}{\sum_{j=1}^{T^{\prime }}\exp\left(e_{j}\right)},\;{\text{a}}_{t}=\alpha_{t}{\tilde{\text{H}}}_{t}. \end{array}$$ (5)

Two bidirectional LSTMs (Hochreiter and Schmidhuber, 1997), with 64 units and sequence output followed by 32 units, integrated the weighted sequence. Dense layers of 64 and 32 units with dropout 0.4 and 0.3 preceded the two-class output:


$$\begin{array}{l} \hat{\text{y}}=\text{softmax}\left({\text{W}}_{o}{\text{Drop}}_{0.3}\left[\text{ReLU}\left({\text{W}}_{32}\text{H}+{\text{b}}_{32}\right)\right]+{\text{b}}_{o}\right). \end{array}$$ (6)

### 3.4 Ablation design

Four prespecified ATCNN-SA variants removed channel attention, temporal attention, both bidirectional LSTMs, or the multi-scale convolutional branches. All remaining layers, optimization settings, folds, and evaluation procedures were held fixed. Because the variants were not retuned independently, these experiments estimate the effect of component removal under a controlled training protocol rather than the best attainable performance of each reduced architecture.

### 3.5 Training, aggregation, and statistical analysis

Configurations were fixed before outer-fold evaluation; no grid, random, or Bayesian search used test participants. Neural models minimized categorical cross-entropy with balanced training-window weights. The three baselines used Adam at 10^−3^, whereas ATCNN-SA used 5 × 10^−4^ (Kingma and Ba, 2015). All models used batch size 128 and at most 60 epochs. Validation loss triggered best-weight restoration after eight unimproved epochs and learning-rate reduction by one half after four unimproved epochs, to a minimum of 10^−6^. The 1D-CNN, CNN-LSTM, EEGNet, and ATCNN-SA contained 47,202, 84,994, 1,730, and 227,667 trainable parameters, respectively.

Window probabilities were summarized for each held-out participant by arithmetic mean, median, normalized geometric mean of class probabilities, and majority vote. A fifth strategy fitted balanced logistic regression (*C* = 0.1) to seven validation-participant summaries: mean, median, geometric probability, majority fraction, standard deviation, and the 25th and 75th percentiles. The trained secondary aggregator was then applied to outer-test participants. A threshold of 0.5 was used after aggregation.

Accuracy, balanced accuracy, precision, sensitivity, specificity, F1, ROC AUC, and precision-recall AUC were computed at window and subject levels. Balanced accuracy was emphasized because the Parkinson's cohort contained twice as many cases as controls. Tables report the mean and sample standard deviation across eight folds. Pooled out-of-fold subject estimates received percentile 95% confidence intervals from 2,000 participant bootstrap samples with replacement (seed 20,265,903). Windows were never resampled as independent observations.

ATCNN-SA was compared with each alternative architecture using two-sided Wilcoxon signed-rank tests on the eight paired fold-wise subject-level balanced accuracies. The three architecture tests within each cohort were Bonferroni-adjusted. Ablation comparisons used the same folds and a separate four-test Bonferroni correction within each cohort. With *n* = 8 and overlapping cross-validation training sets, the tests have limited power and are descriptive rather than confirmatory; nonsignificance is not evidence of equivalence.

### 3.6 Implementation and reproducibility

Experiments ran in Google Colab on Linux with Python 3.13.15, NumPy 2.1.3, pandas 2.2.3, SciPy 1.16.3, scikit-learn 1.6.1 (Pedregosa et al., 2011), MNE 1.12.1, and TensorFlow 2.20.0. The accelerator log identified an NVIDIA A100-SXM4-80GB GPU. Training used float32 arithmetic, cuDNN-compatible recurrent layers with recurrent dropout 0, and XLA/JIT compilation disabled. The archive supplies participant fold assignments, fold-wise metrics, subject out-of-fold probabilities, training histories, timing records, software and protocol records, and the executable analysis script.

## 4 Results

### 4.1 Cross-cohort replication of window leakage

All 121 ADHD participants and 149 Parkinson's participants contributed held-out predictions. The matched audit showed large, concordant leakage effects in both cohorts (Table 2; Figure 2). In ADHD, random-window balanced accuracy was 95.59% ± 0.44%, compared with 60.98% ± 15.07% under participant-disjoint evaluation, a 34.61-percentage-point difference. ROC AUC declined from 0.996 ± 0.001 to 0.663 ± 0.211. Parkinson's showed the same pattern: balanced accuracy decreased from 92.02% ± 0.53% to 56.17% ± 4.33%, a 35.85-point difference, while ROC AUC declined from 0.994 ± 0.001 to 0.675 ± 0.068. Near-perfect random-window performance therefore did not survive evaluation on unseen participants in either cohort.

**Table 2:** Window-level Random Forest performance under contaminated random-window and participant-disjoint evaluation.

Cohort	Split unit	Runs	Acc. (%)	Bal. acc. (%)	ROC AUC
ADHD	Random window	10	96.02 ± 0.41	95.59 ± 0.44	0.996 ± 0.001
	Participant-disjoint	8	62.41 ± 14.04	60.98 ± 15.07	0.663 ± 0.211
Parkinson	Random window	10	94.57 ± 0.34	92.02 ± 0.53	0.994 ± 0.001
	Participant-disjoint	8	68.02 ± 3.18	56.17 ± 4.33	0.675 ± 0.068

Values are run or fold mean ± SD.

**Figure 2:**
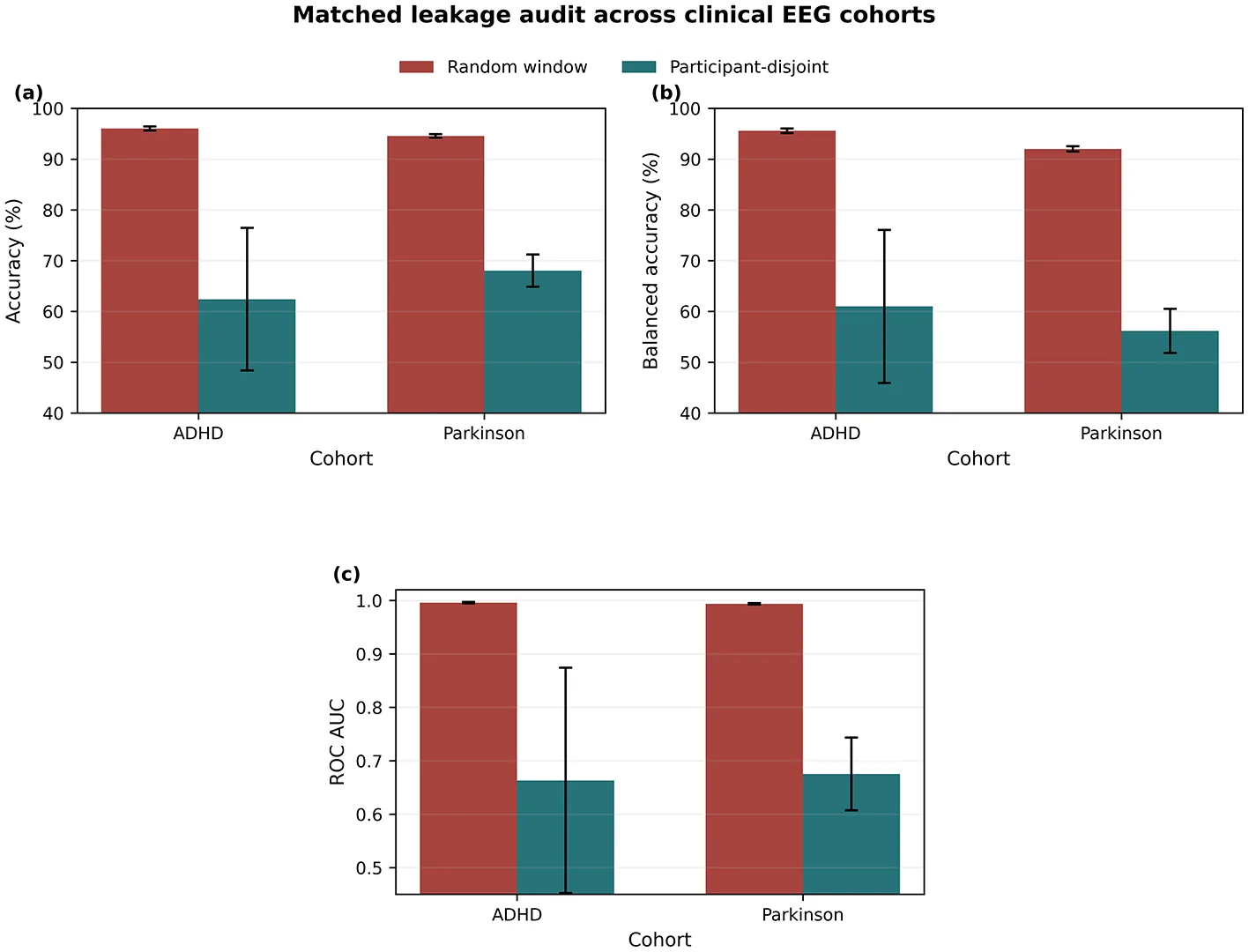
Window-level leakage audit in both cohorts: **(a)** accuracy, **(b)** balanced accuracy, and **(c)** ROC AUC. Bars show means and error bars show ±1 SD across ten random-window repetitions or eight participant-disjoint folds.

This cross-cohort concordance is methodologically informative because the datasets share neither diagnosis nor recording context. ADHD was recorded during a pediatric visual task, whereas the Parkinson's recordings comprise adult eyes-open resting EEG acquired with a denser system. Similar inflation under the same erroneous split points to the segmented participant structure, rather than an ADHD-specific model or acquisition protocol, as the common source. The supplementary data provide the underlying fold and repetition vectors rather than only the aggregate bars.

### 4.2 Subject-level architecture performance

Having established leakage through independent-cohort replication, we next evaluated architecture choice under strict participant separation. Table 3 reports subject-level metrics using mean-probability aggregation. No model dominated both cohorts or all metrics. In ADHD, CNN-LSTM produced the highest mean balanced accuracy (73.21% ± 12.97%) and accuracy (73.33% ± 12.34%), whereas EEGNet produced the highest ROC AUC (0.755 ± 0.184). In Parkinson's, EEGNet produced the highest balanced accuracy (63.75% ± 9.65%) and ROC AUC (0.730 ± 0.083), while 1D-CNN produced the highest conventional accuracy (70.58% ± 8.19%) and F1 (79.12% ± 7.86%). The latter divergence reflects the 100:49 class imbalance and supports balanced accuracy as the primary thresholded metric.

**Table 3:** Subject-level performance under 8-fold participant-disjoint evaluation.

Cohort	Model	Acc. (%)	Bal. acc. (%)	F1 (%)	ROC AUC	PR AUC
ADHD	1D-CNN	59.58 ± 12.14	59.49 ± 12.32	68.15 ± 12.28	0.752 ± 0.159	0.767 ± 0.153
	CNN-LSTM	73.33 ± 12.34	**73.21** **±12.97**	77.13 ± 9.55	0.739 ± 0.187	0.735 ± 0.172
	EEGNet	71.88 ± 11.18	72.66 ± 10.19	65.80 ± 27.38	**0.755** **±0.184**	0.756 ± 0.157
	ATCNN-SA	67.60 ± 15.45	67.86 ± 15.66	68.19 ± 15.28	0.739 ± 0.189	0.728 ± 0.177
Parkinson	1D-CNN	70.58 ± 8.19	62.19 ± 10.41	79.12 ± 7.86	0.687 ± 0.075	0.833 ± 0.044
	CNN-LSTM	64.51 ± 6.98	60.92 ± 7.98	72.09 ± 9.45	0.664 ± 0.098	0.809 ± 0.061
	EEGNet	69.15 ± 6.73	**63.75** **±9.65**	76.96 ± 7.22	**0.730** **±0.083**	0.861 ± 0.046
	ATCNN-SA	66.56 ± 10.19	60.78 ± 6.94	74.03 ± 12.01	0.664 ± 0.084	0.818 ± 0.046

Values are fold mean ± SD using mean-probability aggregation; bold values are the highest balanced accuracy or ROC AUC within each cohort.

Pooled out-of-fold curves in Figure 3 use one probability per participant. Participant bootstrap intervals remained broad despite 15,014 ADHD and 17,056 Parkinson's reference windows. For ADHD CNN-LSTM, pooled balanced accuracy was 0.734 (95% CI: 0.656–0.804) and ROC AUC was 0.749 (0.652–0.834). For Parkinson's EEGNet, pooled balanced accuracy was 0.635 (0.553–0.716) and ROC AUC was 0.694 (0.605–0.781). These intervals are consistent with moderate discrimination and substantial cohort-level uncertainty, not diagnostic readiness.

**Figure 3:**
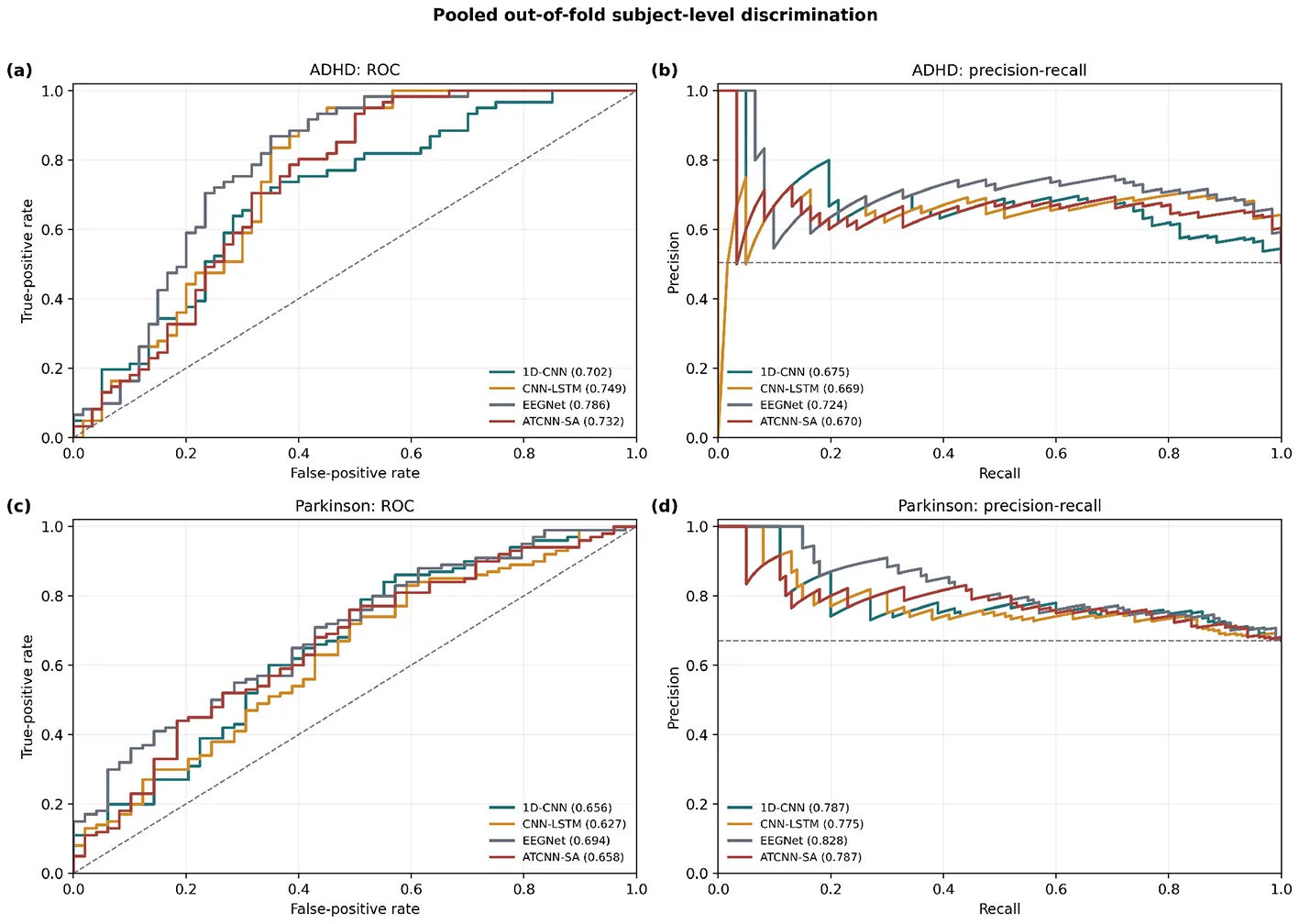
Pooled subject-level discrimination from participant-disjoint out-of-fold predictions: **(a)** ADHD ROC, **(b)** ADHD precision–recall, **(c)** Parkinson's ROC, and **(d)** Parkinson's precision–recall. Each participant contributes one mean probability; legends report pooled AUC.

The paired tests used distinct balanced-accuracy vectors for each comparison. For ADHD, ATCNN-SA versus 1D-CNN, CNN-LSTM, and EEGNet yielded adjusted *p* values of 1.000, 0.891, and 1.000. The corresponding Parkinson's values were all 1.000. Thus, no ATCNN-SA architecture comparison was significant after correction. Fold-level distributions, raw statistics, and participant bootstrap intervals appear in Supplementary Figure S1 and Supplementary Tables S2, S3.

### 4.3 Window duration and subject aggregation

Window duration had no consistent effect across cohorts (Table 4; Figure 4). For the fixed 1D-CNN, ADHD balanced accuracy was highest with 1-s windows (70.98% ± 13.18%), whereas ROC AUC was highest with 8-s windows (0.788 ± 0.190). Parkinson's balanced accuracy was highest at the 2-s reference (62.19% ± 10.41%), but ROC AUC increased descriptively at 4 and 8 s. The 4-s Parkinson's balanced accuracy was 49.60% despite ROC AUC 0.712, indicating threshold and class-balance effects rather than absence of ranking information. Longer windows sharply reduced the number of training examples, from 31,716 to 2,768 in ADHD and from 34,860 to 3,800 in Parkinson's between 1 and 8 s.

**Table 4:** Subject-level 1D-CNN window-duration sensitivity under 8-fold participant-disjoint evaluation.

Cohort	Window (s)	Windows	Bal. acc. (%)	ROC AUC
ADHD	1	31,716	70.98 ± 13.18	0.761 ± 0.192
	2	15,014	59.49 ± 12.32	0.752 ± 0.159
	4	6,754	61.38 ± 13.10	0.772 ± 0.174
	8	2,768	67.41 ± 11.09	0.788 ± 0.190
Parkinson	1	34,860	58.63 ± 5.07	0.668 ± 0.081
	2	17,056	62.19 ± 10.41	0.687 ± 0.075
	4	8,194	49.60 ± 1.13	0.712 ± 0.110
	8	3,800	54.15 ± 6.02	0.715 ± 0.086

Values are fold mean ± SD using mean-probability aggregation.

**Figure 4:**
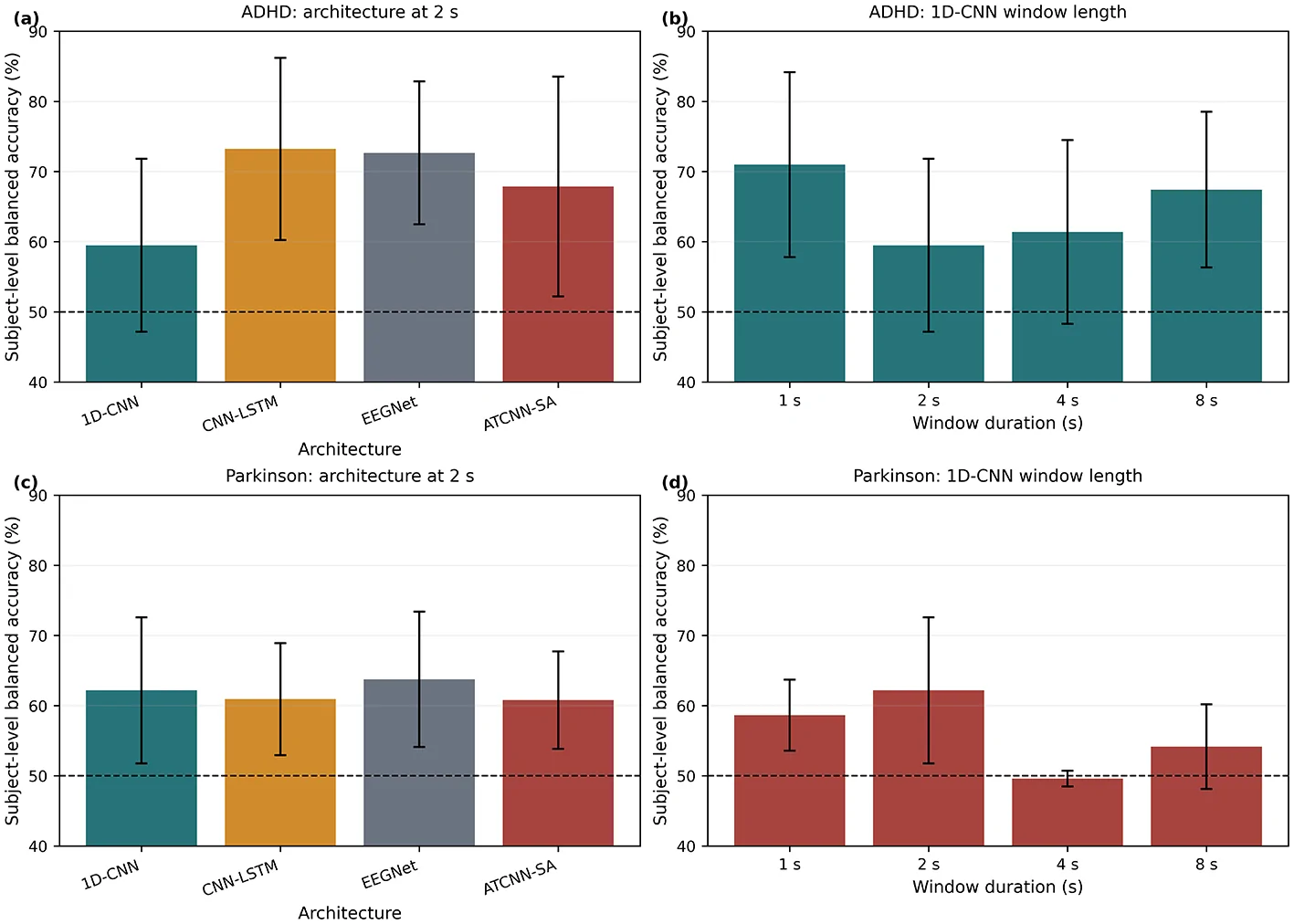
Subject-level balanced accuracy for **(a)** ADHD architectures, **(b)** ADHD window durations, **(c)** Parkinson's architectures, and **(d)** Parkinson's window durations. Bars show eight-fold means and error bars show ±1 SD.

Aggregation changed some participant decisions but did not define a universal best strategy. In ADHD, CNN-LSTM accuracy ranged from 71.72% with geometric aggregation to 75.00% with median or majority-vote aggregation. The validation-trained secondary classifier improved 1D-CNN accuracy from 59.58% to 70.94% but did not improve the other ADHD models consistently. In Parkinson's, secondary classification reduced accuracy for all four architectures relative to mean aggregation. These unstable gains are expected when the secondary classifier is trained on a small inner-validation participant set. All five strategies were retained in Supplementary Table S1 rather than selecting a favorable method after observing test results.

### 4.4 ATCNN-SA component ablations

Component removal produced cohort-dependent patterns (Table 5; Figure 5). In ADHD, removing the bidirectional LSTMs gave the highest ablation balanced accuracy (72.99% ± 9.46%), 5.13 points above full ATCNN-SA; removing temporal attention yielded ROC AUC 0.794 ± 0.139. In Parkinson's, removing temporal attention increased balanced accuracy from 60.78% to 67.22% and ROC AUC from 0.664 to 0.743. Other component removals produced smaller or opposite changes across cohorts. No ablation comparison was significant after four-test Bonferroni correction; the smallest adjusted value was 0.406 for Parkinson's without temporal attention.

**Table 5:** Subject-level ATCNN-SA ablations under 8-fold participant-disjoint evaluation.

Cohort	Variant	Bal. acc. (%)	ROC AUC	Adjusted *p*
ADHD	Full ATCNN-SA	67.86 ± 15.66	0.739 ± 0.189	–
	No channel attention	70.09 ± 14.09	0.752 ± 0.174	1.000
	No temporal attention	71.76 ± 9.64	0.794 ± 0.139	1.000
	No BiLSTM	72.99 ± 9.46	0.759 ± 0.200	1.000
	No multi-scale convolution	65.40 ± 17.23	0.766 ± 0.194	0.969
Parkinson	Full ATCNN-SA	60.78 ± 6.94	0.664 ± 0.084	–
	No channel attention	59.51 ± 7.24	0.685 ± 0.081	1.000
	No temporal attention	67.22 ± 8.99	0.743 ± 0.094	0.406
	No BiLSTM	60.70 ± 9.69	0.701 ± 0.090	1.000
	No multi-scale convolution	59.01 ± 8.20	0.700 ± 0.073	1.000

Metrics are fold mean ± SD using mean-probability aggregation; adjusted *p* values compare balanced accuracy with full ATCNN-SA within cohort.

**Figure 5:**
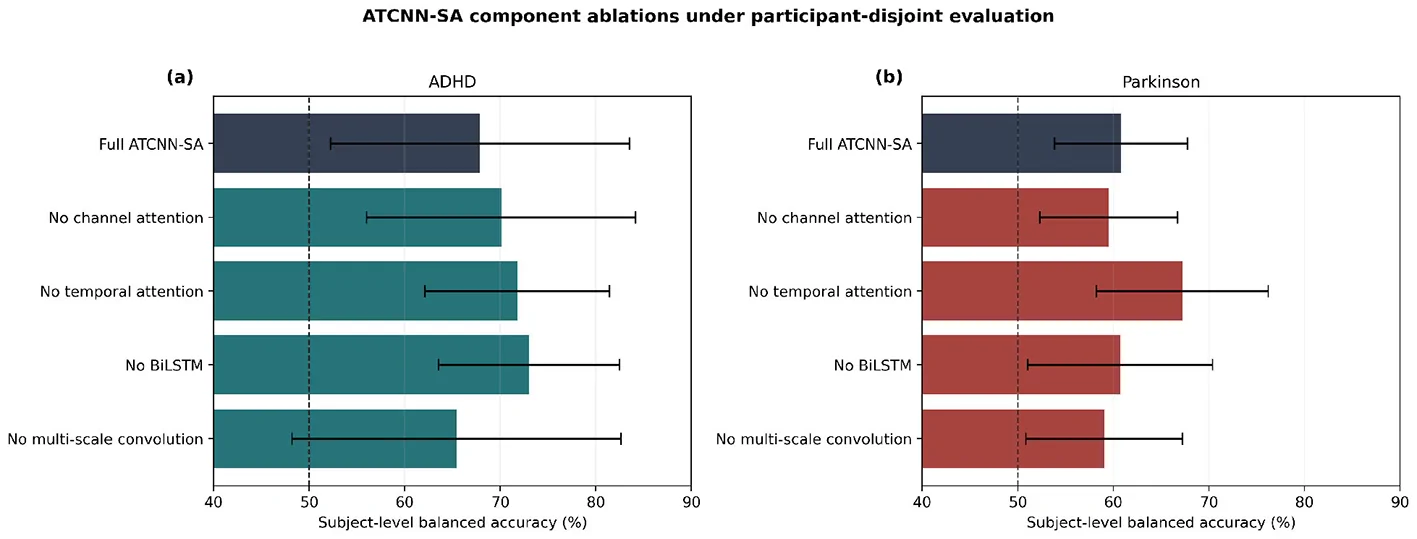
Subject-level balanced accuracy for full ATCNN-SA and four component removals in **(a)** ADHD and **(b)** Parkinson. Bars show 8-fold means and error bars show ±1 SD.

The ablations do not establish that a removed component is harmful or unnecessary. Variants were not retuned independently, and the same eight overlapping cross-validation training sets were reused. The results instead show that the contribution of attention, recurrence, and multi-scale convolution cannot be isolated reliably in cohorts of this size.

### 4.5 Computational cost

Training times were short after recurrent dropout was removed and cuDNN-compatible execution was used (Table 6). Across cohorts, mean per-fold training time ranged from 12.54 to 30.57 s for the 2-s architecture benchmark. ATCNN-SA had the largest parameter count and longest mean training time, while EEGNet had the fewest parameters. Timing values depend on the A100 Colab runtime and should be interpreted as implementation records rather than hardware-independent benchmarks.

**Table 6:** Computational cost for the 2-s architecture benchmark.

Cohort	Model	Parameters	Train/fold (s)	Infer. (ms/window)	Epochs
ADHD	1D-CNN	47,202	21.55 ± 2.62	1.842 ± 0.283	9.1 ± 0.4
	CNN-LSTM	84,994	12.70 ± 1.82	0.349 ± 0.020	10.6 ± 2.1
	EEGNet	1,730	21.25 ± 5.00	1.652 ± 0.137	20.0 ± 11.2
	ATCNN-SA	227,667	29.53 ± 2.62	0.775 ± 0.035	10.5 ± 1.2
Parkinson	1D-CNN	47,202	20.90 ± 1.80	1.359 ± 0.379	9.0 ± 0.0
	CNN-LSTM	84,994	12.54 ± 0.07	0.307 ± 0.024	9.0 ± 0.0
	EEGNet	1,730	17.67 ± 0.65	1.294 ± 0.270	10.8 ± 0.5
	ATCNN-SA	227,667	30.57 ± 2.32	0.693 ± 0.033	9.6 ± 0.9

Values are eight-fold mean ± SD; inference is measured per held-out window.

## 5 Discussion

The central finding is a cross-cohort replication of leakage-driven performance inflation. Random-window balanced accuracy exceeded 92% in both cohorts, yet participant-disjoint balanced accuracy was approximately 61% for ADHD and 56% for Parkinson's with the identical Random Forest pipeline. The decrease was not a peculiarity of one diagnosis, age group, task, channel density, or source sampling rate. Window-level random splitting therefore evaluates recognition of data drawn from recordings already represented during training, not reliable classification of unseen individuals. This result directly supports recent warnings about leakage in translational EEG (Brookshire et al., 2024) and machine-learning-based science more broadly (Kapoor and Narayanan, 2023).

The observed inflation follows directly from the dependence structure of the data. Fifty-percent-overlapping windows share half their samples with adjacent windows, while nonadjacent windows from the same participant retain physiological, acquisition, impedance, session, and behavioral signatures. Random splitting exposes this identity-linked structure on both sides of the evaluation boundary. Treating thousands of windows as independent would also understate uncertainty. The participant-bootstrap intervals remained wide because the effective inferential sample sizes were 121 and 149, not 15,014 and 17,056. Participant separation must therefore precede model fitting and feature selection, and uncertainty must be resampled at the participant level.

Subject-level architecture results reinforce the methodological emphasis. CNN-LSTM led ADHD balanced accuracy, whereas EEGNet led Parkinson's balanced accuracy and ROC AUC. ATCNN-SA was competitive in some metrics but did not dominate either cohort, and no corrected paired comparison was significant. Architecture rankings also differed between conventional accuracy and balanced accuracy in Parkinson's because a classifier could obtain a favorable accuracy and F1 by emphasizing the majority Parkinson's class. Reporting only accuracy would consequently conceal clinically important specificity limitations. Balanced accuracy, sensitivity, specificity, ROC AUC, and PR AUC should be presented together when class prevalence is unequal.

Window duration was similarly dataset- and metric-dependent. Short ADHD windows improved balanced accuracy, while longer ADHD windows produced the highest descriptive ROC AUC. Parkinson's balanced accuracy favored 2 s, whereas longer windows improved AUC without producing a suitable fixed-threshold balance. These results connect windowing to both signal content and sample construction. Short windows provide more training instances but greater redundancy; long windows provide more temporal context but fewer examples. The findings agree with evidence that segmentation and preprocessing can materially alter ADHD EEG classification (García-Ponsoda et al., 2024). They do not identify a universal default beyond requiring the choice to be prespecified and reported.

The component analyses also argue against an architecture-centered claim. Removing recurrence or temporal attention improved descriptive balanced accuracy in one or both cohorts, but the direction and magnitude varied and no corrected comparison was significant. A simplified variant may be easier to optimize in a small cohort, while a larger model may require more data or independent tuning. Because each ablation inherited the full model's hyperparameters, the analysis tests removal under a controlled training protocol rather than the best achievable version of every architecture. Larger preregistered studies should retune each candidate inside nested validation before making component-level causal claims.

Subject aggregation deserves similar restraint. Mean, median, geometric, and majority strategies were often close, but the validation-trained secondary classifier improved one ADHD model and degraded all Parkinson's models. A small validation participant set cannot reliably support another flexible decision layer. Future clinical work should estimate aggregation and operating thresholds using larger development cohorts, assess probability calibration, and evaluate decision-curve or utility-based endpoints rather than selecting aggregation from outer-test performance.

Several limitations bound the conclusions. The two cohorts represent different diseases and recording states; they replicate an evaluation failure mode but do not test cross-disease transfer or external-site generalization within one diagnosis. Harmonization to 18 channels improves comparability but discards ADHD Pz and Parkinson's channels outside the common montage. Parkinson's class imbalance makes thresholded metrics sensitive to the decision rule. Eight outer folds provide only eight paired differences, training sets overlap, and Wilcoxon tests have limited power. Overlapping windows remain correlated within participants even after leakage is prevented. Hyperparameters were fixed rather than selected through a fully nested search, ablations were not independently optimized, and probabilities were not clinically calibrated. The leakage audit used one feature-based classifier; although the mechanism is classifier-independent, the numerical inflation is specific to the present Random Forest and cohorts. Medication state, comorbidities, site effects, and acquisition variables may also contribute residual confounding.

Despite these constraints, the procedural implication is unchanged. A clinically meaningful EEG pipeline must first demonstrate generalization to people who contributed no data to development. Independent cohorts within the same diagnostic target, multi-site evaluation, prespecified preprocessing, nested model selection, calibrated thresholds, and transparent fold files are needed before deployment claims. The supplied fold assignments, out-of-fold probabilities, histories, and executable code make the present estimates auditable and allow future studies to compare methods on the same independent units.

## 6 Conclusion

Window-level random splits contaminated performance in two distinct clinical EEG cohorts. Balanced accuracy fell by 34.61 percentage points in ADHD and 35.85 points in Parkinson's when Random Forest evaluation changed from random windows to unseen participants. This replication makes the practical recommendation unambiguous: do not use random window-level train/test splits to claim clinical generalization.

Under participant-disjoint evaluation, architecture and window rankings depended on cohort and metric, and no ATCNN-SA comparison or component ablation was significant after correction. The models therefore provide moderate research-level discrimination rather than evidence of diagnostic superiority. Practitioners should keep each participant intact throughout development and testing, report subject-level outcomes and class-balanced metrics, resample uncertainty by participant, validate on independent cohorts, and document the exact order of filtering, windowing, splitting, aggregation, and model selection.

## Data Availability

Publicly available datasets were analyzed in this study. The ADHD dataset is available from IEEE DataPort, ‘EEG Data for ADHD/Control Children' (DOI: 10.21227/rzfh-zn36). The Parkinson's disease dataset is available from OpenNeuro, dataset ds004584, version 1.0.0, ‘Rest eyes open' (DOI: 10.18112/openneuro.ds004584.v1.0.0).
