# Supplementary material for: Window leakage in clinical EEG machine learning: matched subject-independent evaluation across ADHD and Parkinson cohorts
Source: Front Neuroinform. 2026 Sep 29;20:1930795. doi: 10.3389/fninf.2026.1930795 (PMC13658158; doi:10.3389/fninf.2026.1930795)
Supplement: Supplementary file 1 [file Supplementary_file_1.zip › frontiers_SupplementaryMaterial.pdf]

Supplementary Material

This supplement accompanies the manuscript *Window Leakage in Clinical EEG Machine Learning: Matched Subject-Independent Evaluation Across ADHD and Parkinson Cohorts*. All displayed values are generated from the accompanying machine-readable files. Window-level observations are correlated segments; subject-level observations contain one aggregated probability per held-out participant. ADHD and Parkinson analyses use the same 18-channel, 128-Hz, 8-fold participant-disjoint protocol.

1 SUPPLEMENTARY TABLES

Supplementary Table S1 compares all five participant-level aggregation rules and shows that no strategy improved both cohorts consistently. Supplementary Table S3 quantifies participant-level uncertainty; the broad intervals remain compatible with moderate discrimination rather than deployment-ready performance. Supplementary Table S5 reports the paired fold comparisons and confirms that no architecture or ablation contrast remained significant after correction. Supplementary Table S7 quantifies the large random-window inflation in both cohorts. Supplementary Table S8 shows that architecture rankings changed across cohorts, and Supplementary Table S9 shows the corresponding dependence on window duration. Supplementary Table S10 documents the cohort-dependent component-removal results, whereas Supplementary Table S11 records model size, training time, inference time, and completed epochs.

Table S1: Subject-level accuracy across five aggregation strategies. Values are eight-fold mean ± SD; each fold contains held-out participants only.

Cohort	Model	Mean	Median	Geometric	Majority	Secondary
ADHD	1D-CNN	59.58 ± 12.14	59.58 ± 12.14	59.58 ± 12.14	59.58 ± 12.14	70.94 ± 11.95
ADHD	CNN-LSTM	73.33 ± 12.34	75.00 ± 12.22	71.72 ± 10.05	75.00 ± 12.22	72.50 ± 12.05
ADHD	EEGNet	71.88 ± 11.18	71.88 ± 11.18	71.88 ± 11.18	71.88 ± 11.18	72.66 ± 13.65
ADHD	ATCNN-SA	67.60 ± 15.45	67.60 ± 14.61	67.60 ± 15.45	67.60 ± 14.61	66.77 ± 15.69
Parkinson	1D-CNN	70.58 ± 8.19	69.30 ± 9.73	70.58 ± 8.19	69.30 ± 9.73	61.81 ± 7.56
Parkinson	CNN-LSTM	64.51 ± 6.98	63.16 ± 6.15	65.86 ± 7.51	63.16 ± 6.15	61.81 ± 11.59
Parkinson	EEGNet	69.15 ± 6.73	68.49 ± 6.48	68.49 ± 8.11	68.49 ± 6.48	59.76 ± 10.36
Parkinson	ATCNN-SA	66.56 ± 10.19	67.21 ± 10.49	66.56 ± 10.19	67.21 ± 10.49	61.73 ± 7.32

Table S3: Participant-bootstrap confidence intervals for pooled subject-level architecture predictions. Each participant contributes one mean probability; intervals use 2,000 bootstrap samples.

Cohort	Model	Metric	Estimate	95% CI low	95% CI high
ADHD	1D-CNN	Accuracy	0.595	0.512	0.686
ADHD	1D-CNN	Balanced accuracy	0.593	0.523	0.663
ADHD	1D-CNN	ROC AUC	0.702	0.603	0.795
ADHD	ATCNN-SA	Accuracy	0.678	0.595	0.760
ADHD	ATCNN-SA	Balanced accuracy	0.677	0.596	0.760
ADHD	ATCNN-SA	ROC AUC	0.732	0.637	0.813
ADHD	CNN-LSTM	Accuracy	0.736	0.653	0.810
ADHD	CNN-LSTM	Balanced accuracy	0.734	0.656	0.804
ADHD	CNN-LSTM	ROC AUC	0.749	0.652	0.834
ADHD	EEGNet	Accuracy	0.719	0.636	0.802
ADHD	EEGNet	Balanced accuracy	0.719	0.637	0.800
ADHD	EEGNet	ROC AUC	0.786	0.696	0.867
Parkinson	1D-CNN	Accuracy	0.705	0.631	0.779
Parkinson	1D-CNN	Balanced accuracy	0.624	0.552	0.697
Parkinson	1D-CNN	ROC AUC	0.656	0.562	0.749
Parkinson	ATCNN-SA	Accuracy	0.664	0.584	0.738
Parkinson	ATCNN-SA	Balanced accuracy	0.609	0.524	0.691
Parkinson	ATCNN-SA	ROC AUC	0.658	0.560	0.752
Parkinson	CNN-LSTM	Accuracy	0.644	0.570	0.725
Parkinson	CNN-LSTM	Balanced accuracy	0.605	0.528	0.689
Parkinson	CNN-LSTM	ROC AUC	0.627	0.532	0.726
Parkinson	EEGNet	Accuracy	0.691	0.611	0.765
Parkinson	EEGNet	Balanced accuracy	0.635	0.553	0.716
Parkinson	EEGNet	ROC AUC	0.694	0.605	0.781

Table S5: Paired Wilcoxon signed-rank tests on subject-level balanced accuracy across eight shared folds. Architecture and ablation families were Bonferroni-adjusted separately within cohort. Positive differences favor full ATCNN-SA.

Cohort	Family	Comparator	W	Raw p	Adjusted p	Δ BA (pp)
ADHD	Architecture	1D-CNN	8.0	0.3750	1.0000	8.37
ADHD	Architecture	CNN-LSTM	7.0	0.2969	0.8906	-5.36
ADHD	Architecture	EEGNet	11.0	0.3750	1.0000	-4.80
ADHD	Ablation	No channel attention	11.0	0.7031	1.0000	-2.23
ADHD	Ablation	No temporal attention	6.0	0.4375	1.0000	-3.91
ADHD	Ablation	No BiLSTM	5.0	0.3125	1.0000	-5.13
ADHD	Ablation	No multi-scale convolution	9.0	0.2422	0.9688	2.46
Parkinson	Architecture	1D-CNN	9.0	0.4688	1.0000	-1.40
Parkinson	Architecture	CNN-LSTM	17.5	0.9844	1.0000	-0.14
Parkinson	Architecture	EEGNet	9.0	0.4688	1.0000	-2.97
Parkinson	Ablation	No channel attention	5.0	0.6250	1.0000	1.28
Parkinson	Ablation	No temporal attention	6.0	0.1016	0.4062	-6.44
Parkinson	Ablation	No BiLSTM	12.0	0.8125	1.0000	0.08
Parkinson	Ablation	No multi-scale convolution	15.0	0.7422	1.0000	1.77

Table S7. Window-level Random Forest leakage audit. Values are mean ± SD across ten random-window repetitions or eight participant-disjoint folds.

Cohort	Split unit	Runs	Acc. (%)	Bal. acc. (%)	ROC AUC
ADHD	Random window	10	96.02 ± 0.41	95.59 ± 0.44	0.996 ± 0.001
	Participant-disjoint	8	62.41 ± 14.04	60.98 ± 15.07	0.663 ± 0.211
Parkinson	Random window	10	94.57 ± 0.34	92.02 ± 0.53	0.994 ± 0.001
	Participant-disjoint	8	68.02 ± 3.18	56.17 ± 4.33	0.675 ± 0.068

Table S8. Subject-level architecture performance using mean-probability aggregation. Values are eight-fold mean ± SD.

Cohort	Model	Acc. (%)	Bal. acc. (%)	F1 (%)	ROC AUC	PR AUC
ADHD	1D-CNN	59.58 ± 12.14	59.49 ± 12.32	68.15 ± 12.28	0.752 ± 0.159	0.767 ± 0.153
	CNN-LSTM	73.33 ± 12.34	**73.21 ± 12.97**	77.13 ± 9.55	0.739 ± 0.187	0.735 ± 0.172
	EEGNet	71.88 ± 11.18	72.66 ± 10.19	65.80 ± 27.38	**0.755 ± 0.184**	0.756 ± 0.157
	ATCNN-SA	67.60 ± 15.45	67.86 ± 15.66	68.19 ± 15.28	0.739 ± 0.189	0.728 ± 0.177
Parkinson	1D-CNN	70.58 ± 8.19	62.19 ± 10.41	79.12 ± 7.86	0.687 ± 0.075	0.833 ± 0.044
	CNN-LSTM	64.51 ± 6.98	60.92 ± 7.98	72.09 ± 9.45	0.664 ± 0.098	0.809 ± 0.061
	EEGNet	69.15 ± 6.73	**63.75 ± 9.65**	76.96 ± 7.22	**0.730 ± 0.083**	0.861 ± 0.046
	ATCNN-SA	66.56 ± 10.19	60.78 ± 6.94	74.03 ± 12.01	0.664 ± 0.084	0.818 ± 0.046

Table S9. Subject-level 1D-CNN window-duration sensitivity using mean-probability aggregation. Values are eight-fold mean ± SD.

Cohort	Window (s)	Windows	Bal. acc. (%)	ROC AUC
ADHD	1	31,716	70.98 ± 13.18	0.761 ± 0.192
	2	15,014	59.49 ± 12.32	0.752 ± 0.159
	4	6,754	61.38 ± 13.10	0.772 ± 0.174
	8	2,768	67.41 ± 11.09	0.788 ± 0.190
Parkinson	1	34,860	58.63 ± 5.07	0.668 ± 0.081
	2	17,056	62.19 ± 10.41	0.687 ± 0.075
	4	8,194	49.60 ± 1.13	0.712 ± 0.110
	8	3,800	54.15 ± 6.02	0.715 ± 0.086

Table S10. Subject-level ATCNN-SA component ablations using mean-probability aggregation. Adjusted p values compare balanced accuracy with full ATCNN-SA.

Cohort	Variant	Bal. acc. (%)	ROC AUC	Adjusted p
ADHD	Full ATCNN-SA	67.86 ± 15.66	0.739 ± 0.189	–
	No channel attention	70.09 ± 14.09	0.752 ± 0.174	1.000
	No temporal attention	71.76 ± 9.64	0.794 ± 0.139	1.000
	No BiLSTM	72.99 ± 9.46	0.759 ± 0.200	1.000
	No multi-scale convolution	65.40 ± 17.23	0.766 ± 0.194	0.969
Parkinson	Full ATCNN-SA	60.78 ± 6.94	0.664 ± 0.084	–
	No channel attention	59.51 ± 7.24	0.685 ± 0.081	1.000
	No temporal attention	67.22 ± 8.99	0.743 ± 0.094	0.406
	No BiLSTM	60.70 ± 9.69	0.701 ± 0.090	1.000
	No multi-scale convolution	59.01 ± 8.20	0.700 ± 0.073	1.000

Table S11. Computational cost for the 2-s architecture benchmark. Times are eight-fold mean ± SD from the recorded A100 runtime.

Cohort	Model	Parameters	Train/fold (s)	Infer. (ms/window)	Epochs
ADHD	1D-CNN	47,202	21.55 ± 2.62	1.842 ± 0.283	9.1 ± 0.4
	CNN-LSTM	84,994	12.70 ± 1.82	0.349 ± 0.020	10.6 ± 2.1
	EEGNet	1,730	21.25 ± 5.00	1.652 ± 0.137	20.0 ± 11.2
	ATCNN-SA	227,667	29.53 ± 2.62	0.775 ± 0.035	10.5 ± 1.2
Parkinson	1D-CNN	47,202	20.90 ± 1.80	1.359 ± 0.379	9.0 ± 0.0
	CNN-LSTM	84,994	12.54 ± 0.07	0.307 ± 0.024	9.0 ± 0.0
	EEGNet	1,730	17.67 ± 0.65	1.294 ± 0.270	10.8 ± 0.5
	ATCNN-SA	227,667	30.57 ± 2.32	0.693 ± 0.033	9.6 ± 0.9

2 SUPPLEMENTARY FIGURES

Supplementary Figure S1 shows substantial between-fold dispersion, particularly in the ADHD cohort, which explains the limited precision of eight-fold comparisons. Supplementary Figure S2 shows that ATCNN-SA required the longest mean training time while EEGNet used the fewest parameters reported in the main text. Supplementary Figure S3 demonstrates the steep reduction in retained segments as window duration increased, without implying a corresponding increase in independent sample size. Supplementary Figure S4 shows stable early stopping across architectures and separates training from inner-validation trajectories; no outer-test participant contributed to these curves.

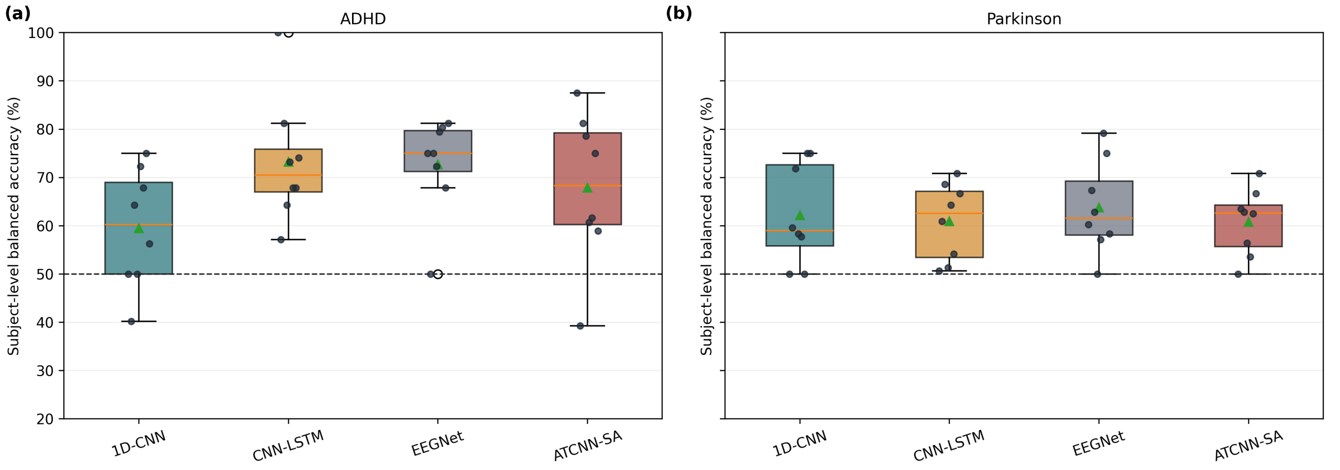

Supplementary Figure S1: Fold-wise subject-level balanced accuracy for the 2-s architecture benchmark in (a) ADHD and (b) Parkinson. Points are participant-disjoint folds; boxes summarize the eight values.

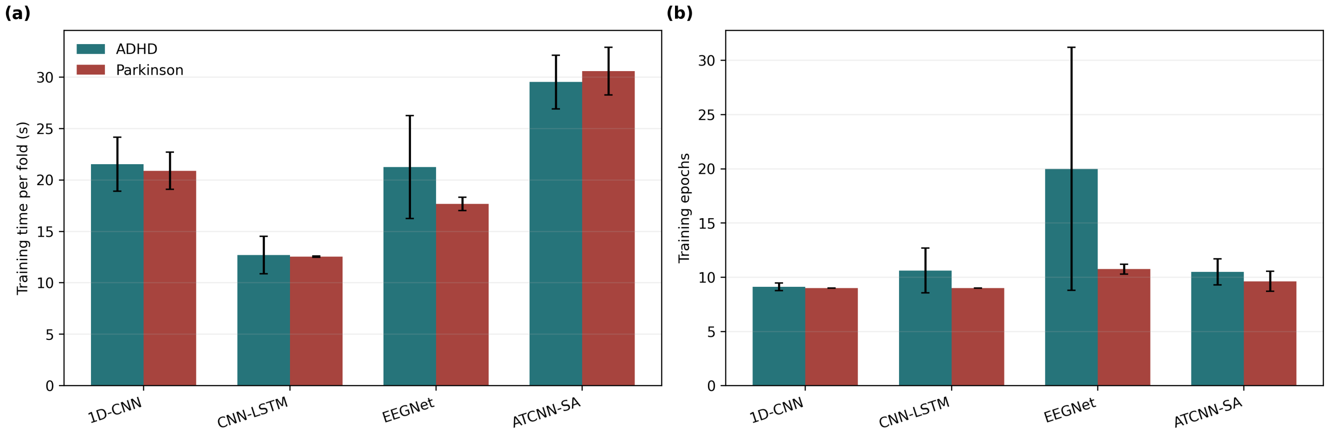

Supplementary Figure S2: Computational behavior of the 2-s architectures: (a) training time per fold and (b) completed optimization epochs. Bars show eight-fold means and error bars show ± 1 SD.

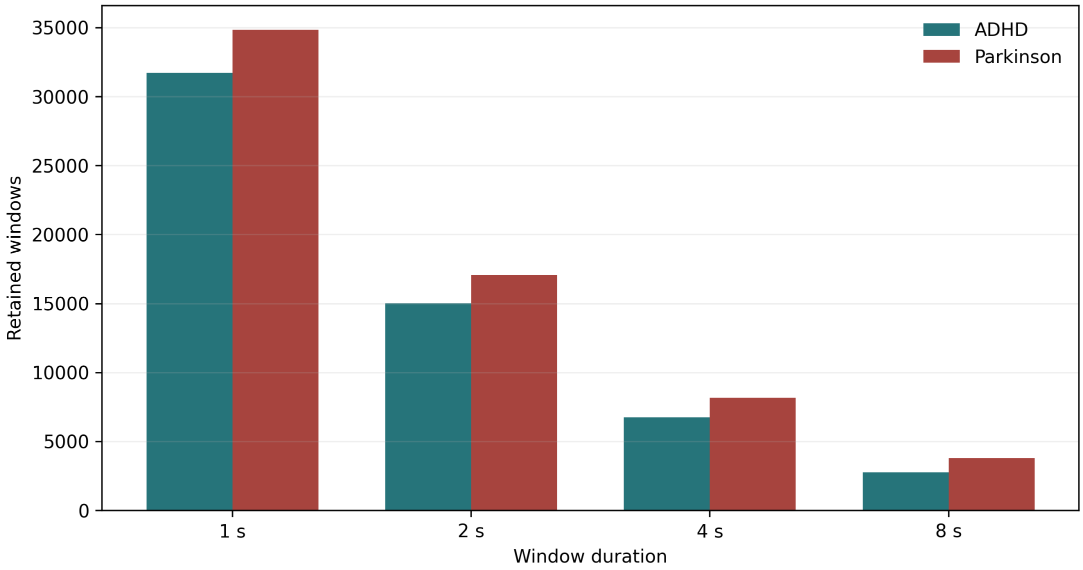

Supplementary Figure S3: Retained window counts after participant-wise preprocessing and the 8-SD artifact rule. Counts are descriptive segments, not independent sample sizes.

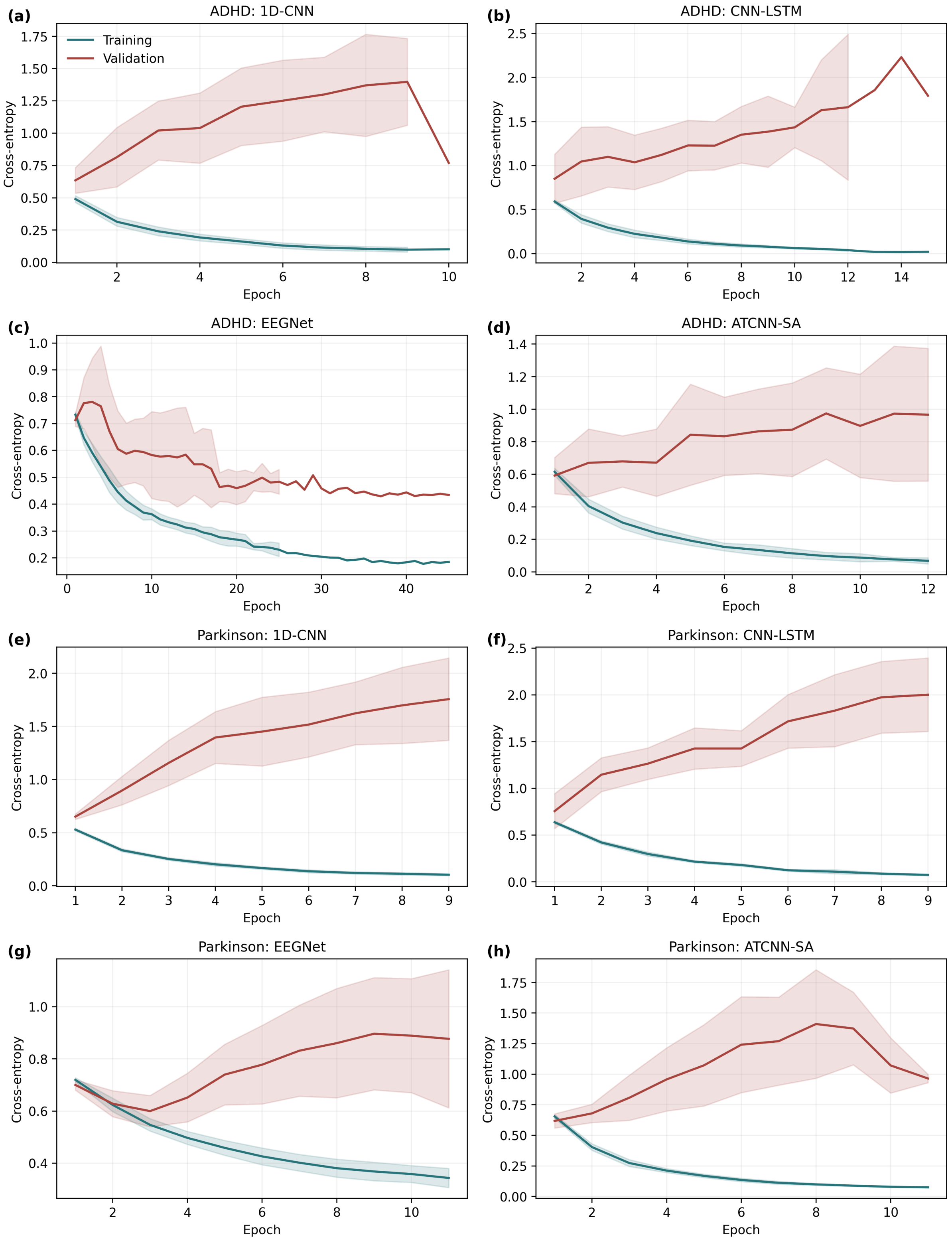

Supplementary Figure S4: Window-level cross-entropy loss for the 2-s architecture benchmark: (a–d) ADHD models and (e–h) Parkinson models, ordered as 1D-CNN, CNN-LSTM, EEGNet, and ATCNN-SA. Lines are fold means and shading is ±1 SD; validation curves contain inner-validation participants only.

3 MACHINE-READABLE SUPPLEMENTARY DATA

The `data` directory contains the complete auditable record of the matched rerun. `fold_assignments.csv` identifies the held-out fold for all 270 participants. `fold_metrics.csv` provides window- and subject-level metrics for every experiment, fold, and aggregation. `subject_oof_predictions.csv` contains the five subject aggregation outputs. `leakage_metrics.csv` provides all eight participant-disjoint and ten random-window Random Forest results per cohort. `paired_wilcoxon_tests.csv` and `subject_bootstrap_intervals.csv` contain the reported inferential summaries. `window_counts.csv`, `training_times.csv`, and the `histories` directory document preprocessing yield and computational behavior. `protocol_config.json`, `run_environment.json`, and `Clinical_EEG_ADHD_Parkinson_CuDNN_Full_Rerun.py` define the analysis environment and executable procedure.
