# Supplementary material for: Window leakage in clinical EEG machine learning: matched subject-independent evaluation across ADHD and Parkinson cohorts
Source: Front Neuroinform. 2026 Sep 29;20:1930795. doi: 10.3389/fninf.2026.1930795 (PMC13658158; doi:10.3389/fninf.2026.1930795)
Supplement: Supplementary file 1 [file Supplementary_file_1.zip › logo1-eps-converted-to.pdf]

frontiers
